# Glycemic control in the context of frailty: a mortality risk assessment in older diabetic patients

**DOI:** 10.1007/s40520-026-03385-5

**Published:** 2026-04-09

**Authors:** Hong-jie Yu, Eric Tsz-Chun Lai, Ruby Yu, Jean Woo

**Affiliations:** 1 Institute of Health Equity, The Chinese University of Hong Kong, Hong Kong SAR, China; 2 Jockey Club Institute of Ageing, The Chinese University of Hong Kong, Hong Kong SAR, China; 3 Department of Medicine & Therapeutics, Faculty of Medicine, The Chinese University of Hong Kong, Hong Kong SAR, China

**Keywords:** Glycemic control, HbA1c levels, Frailty, Mortality risk, Diabetes management

## Abstract

**Background:**

Glycemia control in older diabetic adults is complicated by uncertainty regarding how intensive glycemic control should be, particularly in those with frailty. This study investigated the association between HbA1c levels and all-cause mortality, assessing the modifying effect of frailty.

**Methods/design:**

This retrospective study used Hong Kong hospitalization data (2012–2021) for older adults. Patients with first-recorded diabetes during 2014–2020 were classified as index cases. Frailty was evaluated using the Hospital Frailty Risk Score (HFRS), derived from two years of hospitalization diagnoses before the index diabetes and categorized as follows: no (HFRS = 0), mild (HFRS <5), and moderate andsevere frailty (HFRS ≥5). Glycemic control was assessed via time-weighted mean HbA1c and early variability of the first three measurements. The relationship between HbA1c, variability, and mortality was examined using multivariable Cox regression, with nonlinear relationships modeled via restricted cubic splines.

**Results:**

A J-shaped curve was observed for HbA1c-mortality association (*p*-nonlinearity <0.001), with increased risk at low/high extremes, significantly influenced by frailty (*p*-interaction <0.001). In non-frail patients, lower mortality was noted across an HbA1c range of 5.4%–10.5%, with a minimum at 7.8% (HR = 0.59, 95% CI: 0.56–0.61). For those with moderate andsevere frailty, the safety zone narrowed to 6.1%–9.3%, with a nadir at 7.7% (HR = 0.73, 95% CI: 0.68–0.79). Early glycemic variability significantly affected mortality risk only in non-frail individuals.

**Conclusion:**

Frailty alters the mortality risk associated with glycemic control, highlighting the need for frailty-stratified management strategies in older diabetic adults to optimize health outcomes.

**Supplementary Information:**

The online version contains supplementary material available at 10.1007/s40520-026-03385-5.

## Introduction

The concurrent rise in global population aging and the increasing prevalence of diabetes has led to a rapidly growing population of older adults living with type 2 diabetes (T2D) [1]. Managing T2D in this population presents a more complex clinical challenge, requiring a delicate balance between preventing long-term complications and minimizing the burdens and harms associated with polypharmacy [2, 3]. In contrast to younger patients, older adults with T2D exhibit significant heterogeneity, characterized by considerable variation in physiological reserve and functional capacity, which is influenced by levels of frailty [2, 3]. Within this population, frailty manifests as two distinct phenotypes: anorexic malnourished individuals with low body weight and diminished serum markers, and sarcopenic obese individuals with excessive body weight and elevated glucose and cholesterol levels [4]. This heterogeneity renders uniform glycemic targets inadequate and underscores the necessity for a personalized approach to diabetes care in later life [2, 3].

Although tight glycemic control, defined by a glycated hemoglobin A1c (HbA1c) level below 7%, has been widely recommended to prevent vascular complications [5–7], its application in older adults remains contentious. Current guidelines for older adults propose varying HbA1c targets, typically between 7% and 8.5% [2, 8]. These recommendations largely focused on controlling the risk of hyperglycemia. This perspective is challenged by a persistent epidemiological finding in older adults with diabetes: a J-shaped or U-shaped association between HbA1c and mortality [9–13], wherein both high and low HbA1c levels are linked to increased risk [9]. The excess mortality at lower HbA1c levels is not fully understood, suggesting that conventional unidirectional HbA1c targets may fail to capture critical aspects of patient vulnerability. This risk may represent an unintended consequence of applying intensive control to a biologically heterogeneous aging population, where frailty likely recalibrates the risk–benefit calculus.

Beyond average glycemic exposure, long-term HbA1c variability has emerged as an independent predictor of complications and mortality in diabetes [14–16], potentially reflecting fluctuations in glucose control. In older adults, high HbA1c variability may be an indicator of clinical complexity and dysregulation [17, 18]. Given that frailty represents a state of impaired homeostasis [19], it is plausible that frail individuals possess diminished resilience to the metabolic stresses induced by glycemic fluctuations. Thus, frailty provides a powerful framework for risk stratification among older adults with T2D [20, 21]. However, a critical translational question remains: how can clinicians efficiently identify patients most vulnerable to the harm of intensive glycemic control, particularly in high-risk settings like hospitalization?

The inpatient setting represents a critical environment where frailty, diabetes, and treatment risks intersect, particularly affecting older adults. These individuals are at heightened risk for hypoglycemia, which is associated with increased morbidity, mortality, and healthcare utilization [22–25]. To explore the impact of frailty on treatment variability and risk in this population, our study utilizes the Hospital Frailty Risk Score (HFRS) as an objective stratification tool [26]. The HFRS is a validated, pragmatic tool derived from routinely collected administrative data that quantifies frailty based on a patient's accumulated deficits and clinical complexity during hospitalization [27, 28]. Its selection is supported by distinct advantages, including the ability to be automated and calculated retrospectively for large populations, in contrast to traditional clinical frailty assessments like the Fried Phenotype or the Clinical Frailty Scale, which necessitate prospective, face-to-face evaluations [29]. Thus, this study aims to explore how HFRS-defined frailty moderates the relationship between HbA1c levels and all-cause mortality in older adults hospitalized with T2D. We hypothesize that frailty significantly influences this relationship, anticipating that the J-shaped or U-shaped curve will be more pronounced among frail individuals. Specifically, we expect that their safety HbA1c range, associated with the lowest risk, will be higher compared with that of non-frail counterparts. By elucidating this interaction, our findings seek to inform more precise, person-centered glycemic management strategies for this vulnerable population.

## Methods

### Study design and data source

We conducted a retrospective, territory-wide cohort study utilizing anonymized health records from the Clinical Data Analysis and Reporting System (CDARS), an electronic data repository maintained by Hong Kong’s Hospital Authority (HA). HA oversees the region’s public healthcare services, which encompass 43 hospitals, alongside 49 specialist clinics and 73 general outpatient clinics [30]. As a centralized database, CDARS compiles a wide range of clinical records from all public healthcare facilities in Hong Kong. These records cover patient demographics, mortality data, diagnostic codes, procedural details, prescribed medications, and laboratory findings. The system captures around 80% of all hospital admissions in Hong Kong. The CDARS databases have been rigorously validated, and their recurrent use in significant epidemiological research underscores their reliability for public and clinical health analysis [30, 31]. The study was conducted in accordance with the Declaration of Helsinki. Ethical approval for this research was obtained from the Joint Chinese University of Hong Kong-New Territories East Cluster Clinical Research Ethics Committee (Reference No.: 2022.157). Individual patient informed consent was waived due to the use of anonymized records.

The study period spanned from January 1, 2012, to December 31, 2021. The index period for cohort entry was defined from January 1, 2014, to December 31, 2020, to ensure a minimum two-year pre-index observation period for comorbidity assessment to calculate the HFRS [26] and a minimum one-year post-index follow-up for outcome ascertainment.

### Study population

**Fig. 1:**
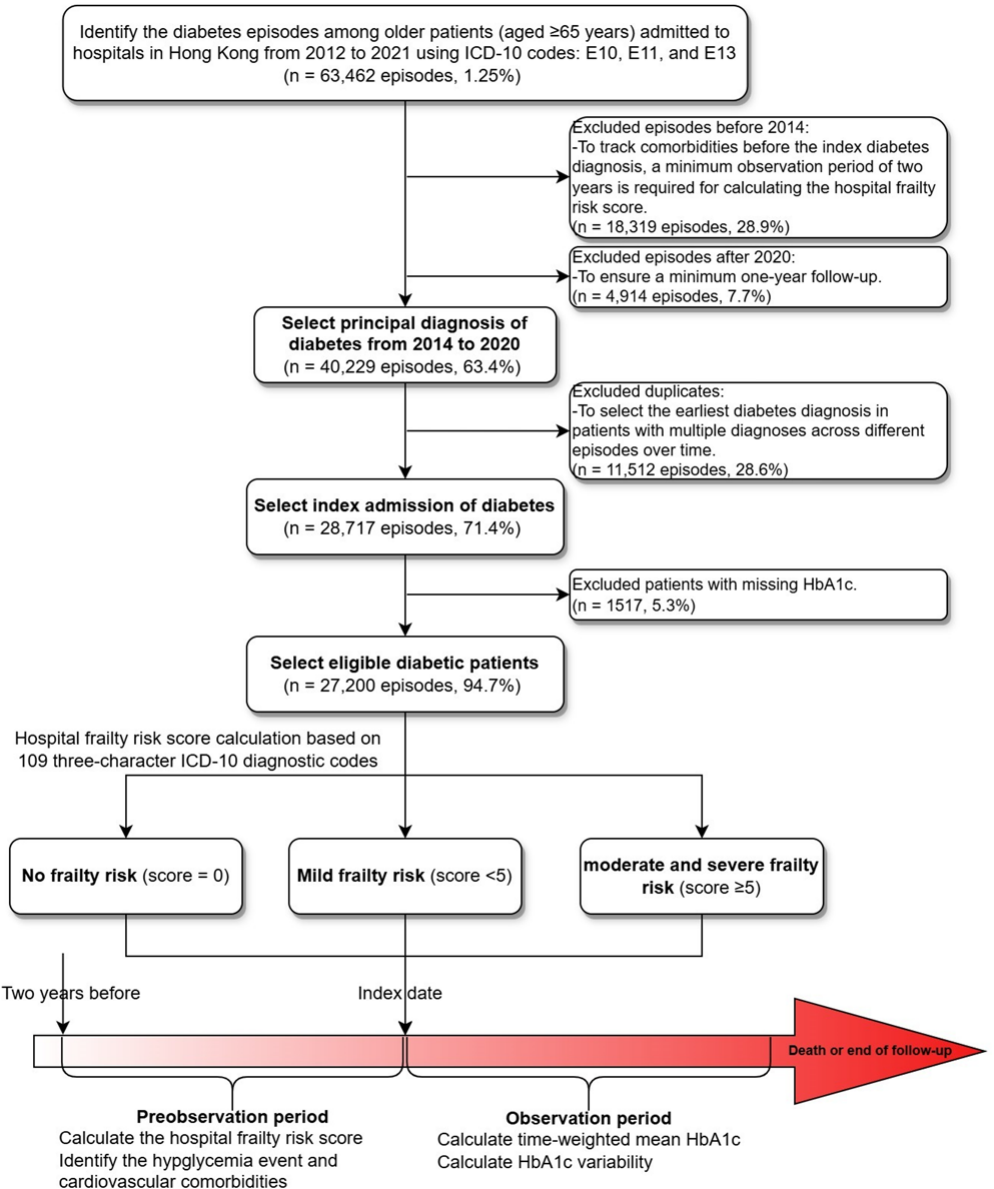
Analysis flowchart

This study identified all hospitalization episodes for patients aged 65 years or older with a primary diagnosis of diabetes mellitus, defined by International Classification of Diseases, Tenth Revision (ICD-10) codes E10, E11, or E13. We excluded patients with type 1 or unspecified diabetes to ensure clinically homogeneous cohort and minimize confounding from differing etiologies and management strategies [32]. The patient selection process followed a staged approach, as illustrated in the accompanying analysis flowchart (Fig. 1). Initially, 63,462 diabetes-related hospitalization episodes were identified between 2012 and 2021. To establish a clear index event, we selected episodes from 2014 to 2020 where diabetes was the principal diagnosis, resulting in 40,229 episodes. For patients with multiple eligible admissions, we selected the earliest admission during the study period as the index admission, yielding 28,717 unique patient episodes. We then excluded 1,517 patients (5.3%) with no recorded HbA1c measurement at any time, resulting in a final analytical cohort of 27,200 patients. The excluded sample exhibited a higher burden of advanced age, old-age home residence, public assistance, pre-index cardiovascular comorbidity, and frailty (Supplementary Table 1).

### Measurements

Glycemic control was evaluated using all available HbA1c measurements (%) recorded from the index date until death or December 31, 2021, whichever occurred first. To characterize clinically relevant, medium-term glycemic patterns while minimizing short-term variability [33], measurements were aggregated into consecutive six-month intervals, yielding interval-specific averages that align with standard diabetes monitoring cycles [34, 35]. The primary exposure was the time-weighted mean HbA1c, calculated by weighting each post-index measurement according to the proportion of follow-up time it represented [33], thereby generating a continuous measure of cumulative glycemic burden that accounts for irregular testing intervals [36]. As a sensitivity analysis, we also examined the overall arithmetic mean of all post-index HbA1c values to assess consistency using a conventional, non-time-weighted metric.

Glycemic variability was assessed using the first three post-index HbA1c measurements to minimize bias from differential follow-up duration and testing frequency. These measurements were derived from the six-month interval averages described in the glycemic exposure assessment. A variability score was calculated by evaluating sequential differences between these three HbA1c values [14–16]. Specifically, we counted the number of adjacent measurement pairs (first–second and second-third) where the absolute difference was ≥ 0.5 percentage points (5.5 mmol/mol) or more, a threshold representing a clinically meaningful change in glycemic control [14–16]. The variability score was expressed as a percentage: (number of changes ≥ 0.5 percentage points/total possible pairs) × 100. Given only two adjacent pairs exist between three measurements, possible scores were 0% (no substantial change, indicating low variability), 50% (one substantial change, indicating moderate variability), or 100% (both pairs showing substantial changes, indicating high variability). Patients with fewer than three post-index HbA1c measurements were excluded from variability analyses.

Frailty was assessed using the HFRS, calculated based on 109 ICD-10 diagnosis codes recorded during the two years preceding the index diabetes admission [26–28]. Diagnoses were mapped to prespecified clinical frailty syndromes, each assigned an empirically derived weight (Supplementary Table 2 [26]), The scoring algorithm focused on serial key clinical constructs associated with frailty, including cognitive impairment, functional dependence, falls and fractures, mood disorders such as anxiety and depression, incontinence, pressure ulcers, and mobility limitations [26]. Weights for each unique diagnosis were summed up to generate a continuous score for each patient. For analysis, patients were categorized into three frailty risk groups: no (HFRS = 0), mild (HFRS < 5), and moderate andsevere frailty risk (HFRS ≥ 5) [27].

The primary outcome was all-cause mortality. Mortality data were obtained through linkage with the hospital records and official Death Registry. Follow-up time was calculated from the index admission date until the date of death or the study end date (December 31, 2021), whichever occurred first. Patients alive at the end of the study were censored at that time.

Baseline covariates included demographic characteristics (age, sex, old-age home residence, and status of recipient of public assistance as a proxy for socioeconomic status) and clinical factors (season of admission and index length of stay). Season of admission was included to account for seasonal variations in disease severity, healthcare utilization [37], and potential environmental influences on glycemic control [38]. Index length of stay served as a marker of acute illness severity and clinical complexity at presentation [39]. We specifically adjusted for pre-existing hypoglycemia (E16.0, E16.1, and E16.2) and cardiovascular disease (CVD, I20.x, I21.x, I22.x, I24.x, I25.x, I46.0, I46.9, I50.x, I11.0, I13.0, I13.2) due to their established associations with both glycemic control and mortality [40, 41]. Hypoglycemia was selected because it represents a treatment-related complication particularly relevant in older adults [20, 25], while CVD is the leading cause of death in diabetes populations [42] and may both confound and modify the relationship between glycemic control and outcomes [40, 41].

### Statistical analysis

Descriptive statistics are presented as medians with interquartile ranges for continuous variables, which displayed skewed distributions, and as frequencies with percentages for categorical variables. Group comparisons across HFRS categories were performed using the Wilcoxon rank-sum test or Kruskal–Wallis test for continuous variables, and the χ^2^ test or Fisher’s exact test for categorical variables, as appropriate.

The association between time-weighted mean HbA1c and mortality was analyzed using multivariable Cox proportional hazards regression. Three sequential models were constructed: Model 1 adjusted only for HFRS category; Model 2 added age, sex, old-age home residence, and payment source; and Model 3 (fully adjusted) further included season of admission and length of stay of index admission, pre-existing cardiovascular disease, and pre-existing hypoglycemia. The proportional hazards assumption was assessed using Schoenfeld residuals.

To examine nonlinearity in the relationship between time-weighted mean HbA1c and mortality, we employed restricted cubic splines with three knots, selected based on improved model fit as indicated by lower Akaike information criterion values. The significance of nonlinearity was tested using analysis of variance. Effect modification by frailty was formally evaluated by testing the interaction between continuous time-weighted mean HbA1c and HFRS category using likelihood ratio tests, comparing models with and without the interaction term. Subgroup analyses were performed by fitting separate Cox models within each HFRS category.

Two additional categorical approaches were used to examine the HbA1c–mortality relationship. First, time-weighted mean HbA1c was grouped according to conventional clinical thresholds: < 7%, 7–8%, 8–9%, and ≥ 9% [9]. Second, using intersection points with hazard ratio (HR) equal to 1, identified from the restricted cubic spline analyses, HbA1c was categorized into three frailty-stratified ranges (low, medium, high) with group-specific cut points that varied by frailty level, reflecting differential safety glycemic targets. In both approaches, Cox models were fitted using the highest HbA1c category as the reference, and interaction with frailty was tested as described above.

After excluding patients with fewer than three six-month HbA1c averages, we examined the association between early glycemic variability and mortality within each frailty stratum. Cox models were fitted with glycemic variability categories (0%, 50%, or 100% variability) as the exposure, adjusted for the same covariates as in the primary analysis, including time-weighted mean HbA1c as a continuous covariate.

Sensitivity analyses examined the robustness of findings by substituting the time-weighted mean HbA1c with the overall arithmetic mean of all post-index HbA1c measurements. Both linear and nonlinear associations between this alternative glycemic metric and mortality were evaluated using the same multivariable Cox and restricted cubic spline modeling frameworks.

All analyses were performed using *R* software (version 4.4.1). A two-sided *p*-value less than 0.05 was considered statistically significant.

## Results

The study cohort consisted of 27,200 older adults with a median age of 78.0 years, of whom 52.9% were female. Stratification by the HFRS revealed that 62.4% of patients (n = 16,966) exhibited no frailty (HFRS = 0), 29.2% (n = 7,954) had mild frailty risk (HFRS < 5), and 8.4% (n = 2,280) were classified as moderate andsevere frailty risk (HFRS ≥ 5). As shown in Table 1, baseline characteristics varied significantly according to frailty status. Individuals with moderate andsevere frailty risk tended to be older (median 83 vs. 76 years), more often female (60.5% vs. 51.0%), more likely to reside in old-age homes (39.4% vs. 6.7%), and more frequently dependent on public assistance (49.1% vs. 30.1%). They also demonstrated a higher prevalence of pre-index hypoglycemia (7.6% vs. 0%) and cardiovascular disease (18.9% vs. 5.7%). With respect to glycemic control, the median of overall mean HbA1c differed significantly across frailty groups (7.68% vs. 7.66% vs. 7.61%; *p*-value < 0.001), whereas time-weighted mean HbA1c showed marginal intergroup variation (7.53% vs. 7.56% vs. 7.50%; *p*-value = 0.051).

**Table 1:** Characteristics of index hospitalization by frailty risk among older adults with diabetes

Characteristics by frailty^*^	**Overall (n = 27,200)**	**No (HFRS = 0, n = 16,966, 62.4%)**	**Mild (HFRS < 5, n = 7954, 29.2%)**	**Moderate and severe, (HFRS ≥ 5, n = 2280, 8.4%)**	***p*-value**^‡^
**Sex**					
Female	14,386 (52.9%)	8659 (51.0%)	4348 (54.7%)	1379 (60.5%)	< 0.001
Male	12,814 (47.1%)	8307 (49.0%)	3606 (45.3%)	901 (39.5%)	
**Age, *years***					
Median [Q1, Q3]	78.0 [71.0, 84.0]	76.0 [70.0, 82.0]	80.0 [74.0, 85.0]	83.0 [77.0, 87.0]	< 0.001
65–74 years	9995 (36.7%)	7337 (43.2%)	2239 (28.1%)	419 (18.4%)	< 0.001
75–84 years	11,180 (41.1%)	6693 (39.4%)	3534 (44.4%)	953 (41.8%)	
**≥ **85 years	6025 (22.2%)	2936 (17.3%)	2181 (27.4%)	908 (39.8%)	
**Old-age home residents**					
No	23,547 (86.6%)	15,835 (93.3%)	6330 (79.6%)	1382 (60.6%)	< 0.001
Yes	3653 (13.4%)	1131 (6.7%)	1624 (20.4%)	898 (39.4%)	
**Season of admission**					
Spring	7386 (27.2%)	4683 (27.6%)	2110 (26.5%)	593 (26.0%)	0.079
Summer	6330 (23.3%)	3983 (23.5%)	1858 (23.4%)	489 (21.4%)	
Fall	6155 (22.6%)	3816 (22.5%)	1816 (22.8%)	523 (22.9%)	
Winter	7329 (26.9%)	4484 (26.4%)	2170 (27.3%)	675 (29.6%)	
**Payment source**					
No public assistance	17,675 (65.0%)	11,865 (69.9%)	4649 (58.4%)	1161 (50.9%)	< 0.001
Public assistance	9525 (35.0%)	5101 (30.1%)	3305 (41.6%)	1119 (49.1%)	
**Length of stay, *days***					
Median [Q1, Q3]	3 [2, 6]	3 [2, 6]	3 [2, 6]	4 [2, 7]	< 0.001
**ICD-10 diagnostic codes**					
E10	177 (0.7%)	118 (0.7%)	48 (0.6%)	11 (0.5%)	0.615
E11	27,023 (99.3%)	16,848 (99.3%)	7906 (99.4%)	2269 (99.5%)	
Hypoglycemia^*^					
No	26,469 (97.3%)	16,966 (100%)	7397 (93.0%)	2106 (92.4%)	< 0.001
Yes	731 (2.7%)	0 (0%)	557 (7.0%)	174 (7.6%)	
Cardiovascular diseases^*^					
No	24,351 (89.5%)	16,004 (94.3%)	6497 (81.7%)	1850 (81.1%)	< 0.001
Yes	2849 (10.5%)	962 (5.7%)	1457 (18.3%)	430 (18.9%)	
**Overall mean HbA1c**^†^**, *%***					
Median [Q1, Q3]	7.67 [6.85, 8.65]	7.68 [6.88, 8.67]	7.66 [6.80, 8.61]	7.61 [6.75, 8.64]	< 0.001
**Time-weighted mean HbA1c**^†^**, *%***					
Median [Q1, Q3]	7.53 [6.74, 8.54]	7.53 [6.77, 8.54]	7.56 [6.73, 8.52]	7.50 [6.66, 8.54]	0.051

Characteristics were presented as n (%) for categorical variables and as median [quartile 1, quartile 3] for continuous variables due to skewed distribution

^*^Frailty risk was evaluated utilizing the Hospital Frailty Risk Score, which considers all hospitalization diagnoses occurring within the two years preceding the index hospitalization. Instances of hypoglycemia and cardiovascular diseases were also identified during this timeframe

^†^Overall mean HbA1c and time-weighted mean HbA1c was calculated by incorporating all available half-year mean HbA1c values recorded since the index hospitalization

^‡^The difference among three frailty status groups were assessed using the Kruskal–Wallis test for continuous variables and Chi-square test for categorical variables, respectively

HFRS: Hospital Frailty Risk Score; ICD-10: International Classification of Diseases, 10th Revision; HbA1c: Hemoglobin A1c

The linear association (Supplementary Table 3) showed that each 1% increase in time-weighted mean HbA1c was associated with a 4.3% increased risk of mortality (95% CI: 1.030–1.056, *p*-value < 0.001) across the entire cohort. Multivariate adjustment for age, sex, residence in old-age home, payment source, season of admission, and length of stay at index admission, and pre-index hypoglycemia and cardiovascular diseases demonstrated minimal confounding on HbA1c-mortality relationship. Notably, this association was significantly modified by frailty. The HR was strongest in patients with no frailty (HR = 1.058, 95% CI: 1.040–1.077, *p*-value < 0.001) and moderate andsevere frailty (HR = 1.060, 95% CI: 1.020–1.101, *p*-value = 0.003), but attenuated and of borderline significance in those with mild frailty risk (HR = 1.022, 95% CI: 1.000–1.045, *p*-value = 0.046).

**Fig. 2:**
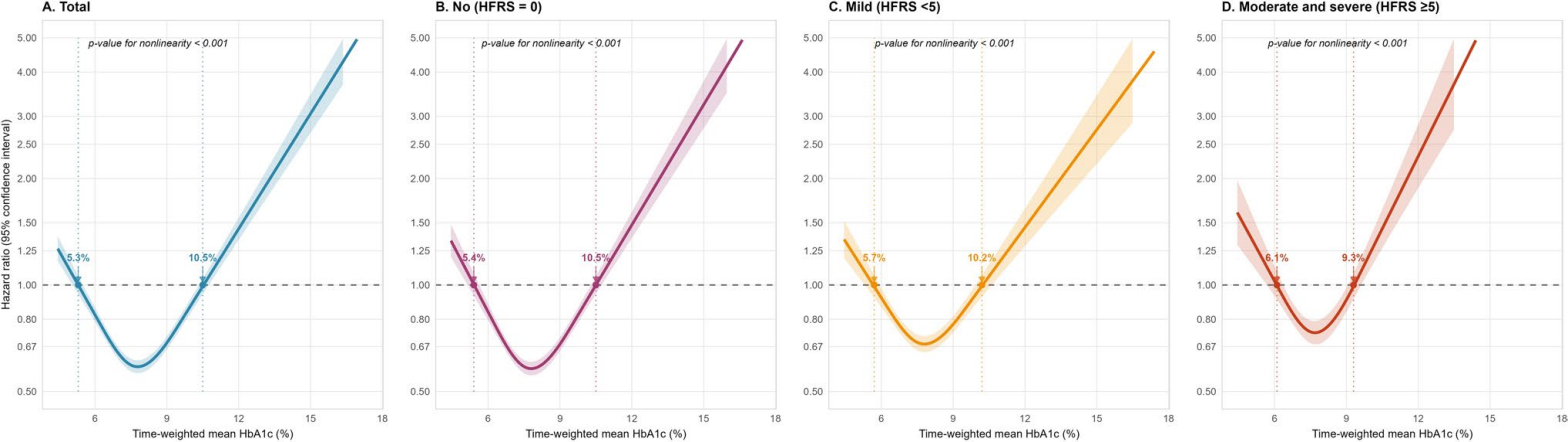
The restricted cubic spline curve for the nonlinear association between time-weighted mean HbA1c and mortality by frailty risk among older adults with diabetes.** A**. Total diabetic patients; **B**. Diabetic patients without frailty risk (HFRS = 0); **C**. Diabetic patients with mild frailty risk (HFRS < 5); **D**. Diabetic patients with moderate and severe frailty risk (HFRS ≥ 5). Restricted cubic spline curve was fitted with three knots at the 10th, 50th, and 90th percentiles using Cox regression. This approach was applied both to the overall population and within specific frailty risk subgroups. The *p*-value for nonlinearity was derived from a Wald Chi-square test. The intersection points on the spline curve represent values of time-weighted mean HbA1c where the hazard ratio equals 1. These points indicate the thresholds at which the predicted mortality risk aligns statistically with the reference risk level. The nadir HbA1c was 7.8% with hazard ratio (HR) 0.59 (95% CI: 0.56–0.61) in total, 7.8% with HR 0.58 (95% CI: 0.56–0.61) in no frailty, 7.8% with HR 0.68 (95% CI: 0.65–0.72) in mild frailty, and 7.7% with HR 0.73 (95% CI: 0.68–0.79) in moderate and severe frailty The Cox regression model was adjusted for a variety of covariates, including age, sex, residence in old-age home, payment source, season of admission, length of stay during the index hospitalization, and instances of hypoglycemia and cardiovascular events occurring within two years prior to the index hospitalization. Frailty risk was evaluated utilizing the Hospital Frailty Risk Score (HFRS), which considers all hospitalization diagnoses occurring within the two years preceding the index hospitalization. Instances of hypoglycemia and cardiovascular diseases were also identified during this timeframe

Restricted cubic spline analysis revealed a nonlinear relationship between time-weighted mean HbA1c and mortality (*p*-value for nonlinearity < 0.001), with significant variation by frailty status (Fig. 2). In both the overall cohort and among patients without frailty, the relationship between HbA1c levels and mortality exhibited a J-shaped curve, with the lowest mortality risk (HR = 0.59; 95% CI: 0.56–0.61) observed at approximately 7.8% HbA1c. Elevated mortality risk was noted at values below 5.4% and above 10.5%. Among individuals with mild or moderate to severe frailty, the glycemic range associated with lower mortality risk progressively narrowed; the two points at which the HR equaled 1 converged toward the median, while the mortality hazard at the nadir was diminished, despite a similar corresponding HbA1c level. In the moderate and severe frailty groups, a statistically significant increase in mortality risk was observed at HbA1c levels below 6.1% and above 9.3%, with the lowest HR of 0.73 (95% CI: 0.68–0.79) occurring at an HbA1c level of 7.7%.

Using conventional clinical categories (Table 2), an HbA1c < 7% was associated with lower mortality compared with > 9% in all frailty groups (e.g., No frailty: HR = 0.878, 95% CI: 0.819–0.941; Mild: HR = 0.902, 95% CI: 0.829–0.981; Moderate and severe: HR = 0.777, 95% CI: 0.674–0.897). However, when using frailty-stratified thresholds derived from the spline curves, the association patterns differed markedly (*p*-value for interaction < 0.001). In the no frailty group, an HbA1c range of 5.4–10.5% was associated with a 50% lower mortality risk (HR = 0.499, 95% CI: 0.452–0.551) compared with the “High” group (> 10.5%). A similar but less pronounced pattern was seen in the mild frailty group (range 5.7–10.3%: HR = 0.624, 95% CI: 0.553–0.703). In the moderate andsevere frailty group, a narrower range of 6.1–9.3% was associated with a 31% lower risk (HR 0.685, 95% CI 0.591–0.793) compared with > 9.3%. No significant benefit in mortality risk was observed for the “Low” HbA1c group compared with the “Medium-range” HbA1c group.

**Table 2:** Association between different HbA1c groups and mortality by frailty risk among older adults with diabetes

**Frailty risk**	HbA1c group^*^	**N**	**Death**	**Person- years**	**Mortality (per 100 person-years)**	**Hazard ratio (95% confidence interval)**	***p*-value**	***p*-value for interaction**^†^
**Conventional HbA1c classification**								0.302
No (HFRS = 0)	> 9%	2981	1281	3196.22	40.08	Reference		
No (HFRS = 0)	8 to 9%	3242	1310	4145.22	31.60	0.750 (0.694–0.810)	< 0.001	
No (HFRS = 0)	7 to 8%	5225	1937	6305.46	30.72	0.706 (0.657–0.758)	< 0.001	
No (HFRS = 0)	< 7%	5518	2340	6542.27	35.77	0.878 (0.819–0.941)	< 0.001	
Mild (HFRS < 5)	> 9%	1326	809	1844.77	43.85	Reference		
Mild (HFRS < 5)	8 to 9%	1664	995	2747.22	36.22	0.786 (0.716–0.862)	< 0.001	
Mild (HFRS < 5)	7 to 8%	2336	1370	3883.27	35.28	0.767 (0.703–0.838)	< 0.001	
Mild (HFRS < 5)	< 7%	2628	1743	4291.29	40.62	0.902 (0.829–0.981)	0.016	
Moderate and severe (HFRS ≥ 5)	> 9%	386	291	531.49	54.75	Reference		
Moderate and severe (HFRS ≥ 5)	8 to 9%	453	336	757.75	44.34	0.751 (0.641–0.879)	< 0.001	
Moderate and severe (HFRS ≥ 5)	7 to 8%	627	441	1117.44	39.47	0.665 (0.572–0.774)	< 0.001	
Moderate and severe (HFRS ≥ 5)	< 7%	814	607	1356.87	44.74	0.777 (0.674–0.897)	0.001	
**HbA1c group defined based on restricted cubic spline curve**								< 0.001
No (HFRS = 0)	High (> 10.5%)	943	432	780.11	55.38	Reference		
No (HFRS = 0)	Medium (5.4 to 10.5%)	15,798	6275	19,121.25	32.82	0.499 (0.452–0.551)	< 0.001	
No (HFRS = 0)	Low (< 5.4%)	225	161	287.81	55.94	0.988 (0.824–1.186)	0.899	
Mild (HFRS < 5)	High (> 10.2%)	458	287	538.04	53.34	Reference		
Mild (HFRS < 5)	Medium (5.7 to 10.2%)	7131	4342	11,673.87	37.19	0.624 (0.553–0.703)	< 0.001	
Mild (HFRS < 5)	Low (< 5.7%)	365	288	554.63	51.93	0.953 (0.809–1.124)	0.570	
Moderate and severe (HFRS ≥ 5)	High (> 9.3%)	284	213	374.51	56.87	Reference		
Moderate and severe (HFRS ≥ 5)	Medium (6.1 to 9.3%)	1744	1261	3050.89	41.33	0.685 (0.591–0.793)	< 0.001	
Moderate and severe (HFRS ≥ 5)	Low (< 6.1%)	252	201	338.15	59.44	1.049 (0.863–1.274)	0.632	

^*^The HbA1c groups were defined using two methods: a conventional classification and a restricted cubic spline curve for the time-weighted mean HbA1c. The association between these groups and mortality was assessed using Cox regression analysis, adjusting for age, sex, residence in an old-age home, payment source, season of admission, length of stay, and occurrences of hypoglycemia and cardiovascular events within the two years prior to hospitalization

^†^The *p*-value for interaction was assessed using a likelihood ratio test, which compared Cox regression models with and without the interaction term for HbA1c group and frailty risk group

Frailty risk was evaluated utilizing the Hospital Frailty Risk Score (HFRS), which considers all hospitalization diagnoses occurring within the two years preceding the index hospitalization. Instances of hypoglycemia and cardiovascular diseases were also identified during this timeframe

The association between early HbA1c variability and mortality demonstrated notable variation by frailty status (Table 3), although the interaction was not statistically significant (*p*-value for interaction = 0.519). Among patients with no frailty, both 50% and 100% variability were associated with significantly increased mortality risk compared with 0% variability (HR = 1.162, 95% CI: 1.061–1.272 and HR = 1.106, 95% CI: 1.010–1.210, respectively). In contrast, no significant associations were observed among those with mild frailty or moderate andsevere frailty.

**Table 3:** Association between HbA1c variability quartile and mortality by frailty risk among older adults with diabetes

**Frailty risk**	Variability^*^	**N**	**Death**	**Person-years**	**Mortality (per 100 person-years)**	**Hazard ratio (95% confidence interval)**	***p*-value**
No (HFRS = 0)	0%	2281	674	2840.97	23.72	Reference	
No (HFRS = 0)	50%	5301	2040	7858.89	25.96	1.162 (1.061–1.272)	0.001
No (HFRS = 0)	100%	5490	1690	6757.89	22.01	1.106 (1.010–1.210)	0.029
Mild (HFRS < 5)	0%	816	369	1404.57	26.27	Reference	
Mild (HFRS < 5)	50%	2378	1310	4753.25	27.56	1.102 (0.978–1.241)	0.112
Mild (HFRS < 5)	100%	2296	1188	4395.13	27.03	1.073 (0.953–1.207)	0.245
Moderate and severe (HFRS ≥ 5)	0%	160	83	300.57	27.61	Reference	
Moderate and severe (HFRS ≥ 5)	50%	605	388	1381.81	28.08	0.961 (0.751–1.230)	0.751
Moderate and severe (HFRS ≥ 5)	100%	547	352	1232.55	28.56	1.027 (0.804–1.312)	0.831

^*^HbA1c variability was calculated as the percentage of instances in which successive HbA1c values changed by 0.5% relative to the total number of comparisons among the first three mean HbA1c values. The association between these groups and mortality was assessed using Cox regression analysis, adjusting for time-weighted mean HbA1c, age, sex, residence in an old-age home, payment source, season of admission, length of stay, and occurrences of hypoglycemia and cardiovascular events within the two years prior to hospitalization

Frailty risk was evaluated utilizing the Hospital Frailty Risk Score (HFRS), which considers all hospitalization diagnoses occurring within the two years preceding the index hospitalization. Instances of hypoglycemia and cardiovascular diseases were also identified during this timeframe

The *p*-value for the interaction between frailty risk and HbA1c variability is 0.519. This was assessed using a likelihood ratio test that compared Cox regression models with and without the interaction term for HbA1c group and frailty risk group

Sensitivity analysis using the overall mean HbA1c value to repeat the linear (Supplementary Table 4) and nonlinear association (Supplementary Fig. 1) with mortality showed similar results.

## Discussion

In this large retrospective cohort study of older adults with diabetes in Hong Kong, we identified a J-shaped association between the time-weighted mean HbA1c and all-cause mortality, with the lowest risk observed at approximately 7.8%. Critically, we demonstrate that this relationship is fundamentally modified by frailty status. The glycemic range associated with a reduced mortality risk contracts from 5.4–10.5% in non-frail individuals to only 6.1–9.3% in those with moderate andsevere frailty. Furthermore, the increased mortality risk associated with early glycemic variability was confined to non-frail individuals. These findings challenge the application of uniform treatment targets and suggest the potential value of personalized, frailty-stratified approaches to diabetes management in older adults.

Our observation of a J-shaped relationship between HbA1c and mortality aligns with existing literature focused on older and clinically complex population [9–11, 13, 33, 43]. Studies of older adults with insulin-treated diabetes [9], as well as those with heart failure [33] and coronary artery disease [11], have consistently identified elevated mortality risk at both lower and higher glycemic extremes. For example, harmful ranges have been reported as ≤ 6.5% and > 11.5% versus a reference of 6.5–7.4% [9], ≤ 7% and > 9% versus 7–9% [33], and ≤ 5.7% and > 6.7% versus 5.7–6.1% [11]. Two studies also utilized restricted cubic spline analysis to model this nonlinear relationship continuously [10, 11], identifying nadirs of mortality risk at 5.7% [11] and 6.9% [10], which is notably lower than the 7.8% nadir in our study. These inconsistencies in reported nadirs of mortality risk are potentially due to our specific focus on hospitalized older adults with diabetes, contrasting with the broader populations targeted in community setting [10, 11]. Additionally, subgroup analyses in two studies suggested that the adverse effect of lower HbA1c was more pronounced in advanced age groups (≥ 75 [11] or ≥ 70 [43] years). Although these prior works correctly highlight the dangers of intensive glycemic control in high-risk patients, their risk stratification has predominantly relied on chronological age or the presence of specific comorbidities.

Our analysis utilized frailty, a direct, integrative measure to assess biological aging and physiological reserve [21, 44] to reframe the central clinical question from “How old is the patient?” to “How resilient is this patient?” We demonstrated that frailty modifies the association between HbA1c and mortality. The progressive attenuation and narrowing of the J-shaped relationship with increasing frailty burden provides a crucial lens through which to interpret prior, sometimes seemingly conflicting, epidemiological data [13, 45]. In patients with moderate andsevere frailty, the HbA1c range, associated with a lower mortality, contracted to 6.1–9.3%, and the characteristic elevation in risk associated with moderate hyperglycemia (the right arm of the J-curve, from 9.3% to 10.4%) was no longer statistically significant. This transformation suggests that the physiological reserve to tolerate metabolic derangements may be depleted in frailty [19]. The loss of a protective buffer at higher HbA1c levels may reflect a diminished capacity to withstand the catabolic, pro-inflammatory, and osmotic stresses of hyperglycemia [3, 6]. Concurrently, the persistent significant risk at the lower glycemic extreme underscores a heightened vulnerability to hypoglycemia [43, 46, 47], likely exacerbated by polypharmacy, anorexia, and renal impairment common in frail individuals [25, 46]. This gradient explains why studies conducted in healthier older cohorts may still detect a U-curve, while those in sicker, frailer populations often report a more linear or J-shaped association [40, 45, 48].

By demonstrating that the range of relative glycemic safety narrows predictably with increasing frailty severity, we provide a more precise, empirically grounded framework for personalizing diabetes care in older adults. This directly addresses a critical gap highlighted in recent systematic reviews of guidelines for older and frail individuals [8], which consistently recommend relaxed targets (e.g., < 8.0% or < 8.5%) for patients with significant comorbidity or limited life expectancy, yet acknowledge a persistent lack of precision and evidence-based thresholds [8]. The present analysis moves beyond consensus by quantifying how the risk–benefit profile changes with frailty. However, the clinical application of these statistically derived ranges requires careful interpretation. These intervals represent the statistical “safety zone” where mortality risk is not significantly elevated relative to the nadir [10, 11]. The primary clinical utility of these findings lies less in their absolute breadth than in their systematic narrowing with advancing frailty.

The analysis of HbA1c variability further refines this frailty-stratified model. The significant association between increased variability (both 50% and 100% increases) and higher mortality in the non-frail group underscores that glycemic instability is an independent risk factor in relatively healthy older adults, possibly indicating poor treatment adherence or underlying metabolic lability [14–16]. The lack of a significant association in both the mild and moderate andsevere frailty groups is a critical finding. It may indicate that in these populations, the overwhelming risk is conferred by the presence and severity of frailty syndromes and comorbidities, such that the additional hazard from glucose fluctuation is marginal [49]. Alternatively, it could suggest that the glycemic profiles of frail patients are already more tightly managed or less variable due to greater clinical supervision [50], or that shorter life expectancy precludes the observation of long-term consequences of variability.

### Strengths and limitations

The strengths of this study include its large, real-world cohort; the use of a validated, administrated-based HFRS that allows for retrospective frailty risk stratification [26, 27]; and the application of time-weighted mean HbA1c to better represent long-term glycemic exposure [33]. The use of nonlinear modeling was essential to accurately characterize the mortality risk relationship. This assessment is especially pertinent for Hong Kong’s rapidly aging population. Local research has indicated that frailty is not only prevalent [51, 52], but its trajectories show complex sociometric patterns, and it is a leading attributable cause of functional decline and hospitalization [51]. In a system where acute hospital beds are a critical resource, hypoglycemic events or hyperglycemic crises in a frail older adult can trigger a cascade of iatrogenic complications, prolonged stays, and irreversible loss of independence [24]. Therefore, passively relaxing targets are insufficient. Proactive, systematic frailty screening in diabetes clinics should trigger a fundamental care-plan review. This strategy aligns with local evidence supporting integrated care models for community-dwelling frail older people. Implementing such models with embedded, frailty-stratified diabetes protocols, enabling collaboration between endocrinologists, geriatricians, and community nurses, could enhance safety [51]. The objective shifts from aggressive glycemic control to maintaining stability within a personalized, physiology-congruent range to preserve function and quality of life.

Several limitations must be considered when interpreting these findings. First, regarding study design and population, this was a retrospective, observational analysis using administrative data such that the observed relationships might not be causal. The cohort was identified through hospital admissions, likely selecting individuals with greater healthcare needs or acute morbidity; this may limit the generalizability of our findings to healthier, entirely community-dwelling older adults. Moreover, the study was conducted within the specific context of Hong Kong's public healthcare system and its predominantly Chinese older population. The applicability of these frailty-stratified thresholds to other ethnic groups [53] or healthcare delivery models requires external validation. Second, the assessment of frailty using the HFRS, while validated and pragmatic for large-scale research [26–28], is an administrative proxy that may not fully capture the multidimensional clinical reality of frailty as assessed by tools like the Clinical Frailty Scale [29], potentially leading to misclassification. Furthermore, the timing and frequency of HbA1c measurements were dictated by clinical care rather than a standardized research protocol. To address this inherent variability and potential gaps in the longitudinal glycemic profile, we employed and cross-validated two complementary exposure measures, time-weighted mean HbA1c and overall mean HbA1c, which yielded consistent results, strengthening confidence in our primary findings. Third, unmeasured and residual confounding cannot be ruled out. Most notably, our dataset lacked information on specific glucose-lowering medications (e.g., metformin, sulfonylureas, insulin, dipeptidyl peptidase 4 inhibitors, glucagon-like-peptide 1 receptor agonists, and sodium glucose co-transporter-2 inhibitors [54, 55]), some of which confer greater hypoglycemia risk, such as insulin compared with sulfonylureas [46]. The inability to adjust for these differential effects means the observed HbA1c-mortality association could be confounded by therapy type. However, recent local data from Yang et al. (2022) provide important context: despite increased use of newer agents associated with lower hypoglycemia risk in Hong Kong, rates of severe hypoglycemia requiring hospitalization did not decline in older adults, instead plateauing [55]. This potentially suggests that patient-level factors, particularly frailty, may be more dominant drivers of adverse outcomes than drug class alone. Other unmeasured clinical details, including nutritional status [4, 20, 56], serial renal function, cognitive impairment, and community-managed hypoglycemia [46], were also unavailable for adjustment. Furthermore, our primary outcome was all-cause mortality; we did not examine cause-specific mortality or the incidence of diabetes-related complications, particularly cardiovascular events [48]. The HbA1c range associated with the lowest risk of complications may differ from the range optimal for survival [42, 43, 48], a nuance our study could not explore. Accordingly, these findings should be viewed as hypothesis-generating and provide a strong rationale for future research incorporating linked pharmacy data, prospective frailty assessments, and detailed comorbidity profiles to validate and extend our observations.

## Conclusion

The risk associated with glycemic control in older adults with diabetes is contingent upon physiological resilience, as measured by frailty. Frailty transforms the risk profile, narrowing the safe glycemic range and obscuring the impact of glucose variability on mortality risk. Integrating routine frailty assessment into diabetes care to guide personalized, stability-focused management is a critical step toward mitigating preventable harm in a vulnerable, growing population. This approach aligns the goals of diabetes therapy with the overarching priority of geriatric care: to maintain function and quality of life.

## Supplementary Information

Below is the link to the electronic supplementary material.

Supplementary file1 (JPG 1005 kb)

Supplementary file2 (DOCX 381 kb)

## Data Availability

The datasets for the current study are available from the corresponding author upon reasonable request, contingent upon receiving approval from the Hong Kong Hospital Authority.
