# Supplementary figures and images for: Glycemic control in the context of frailty: a mortality risk assessment in older diabetic patients
Source: Aging Clin Exp Res. 2026 Apr 9;38(1):129. doi: 10.1007/s40520-026-03385-5 (PMC13194240; doi:10.1007/s40520-026-03385-5)

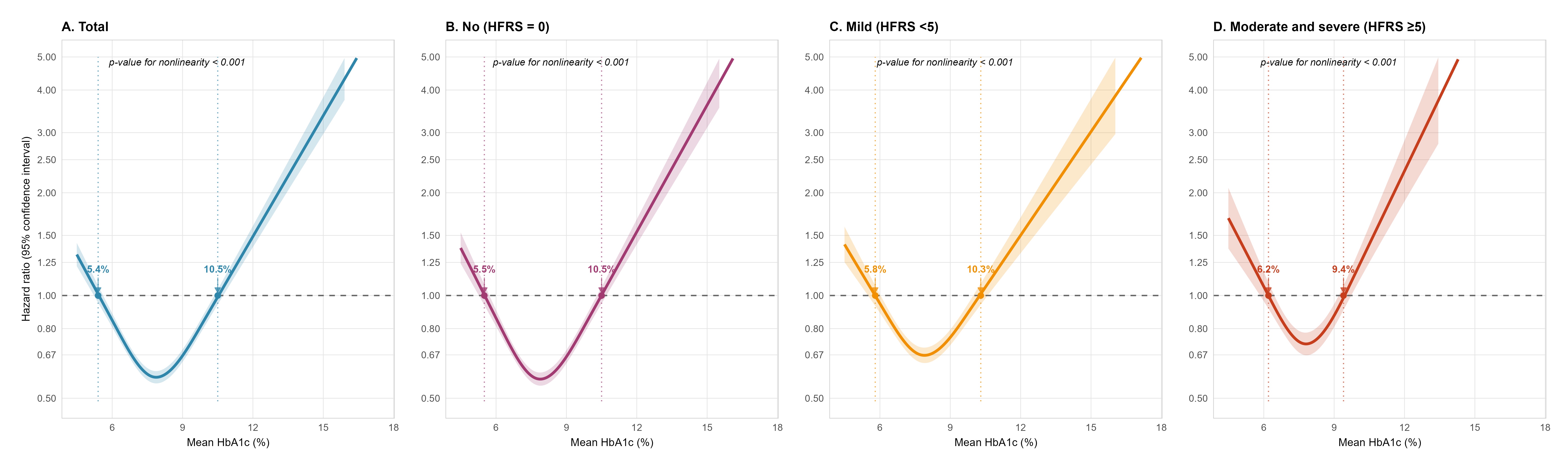

Supplement: Supplementary file 1 — Supplementary file1 (JPG 1005 kb) [file 40520_2026_3385_MOESM1_ESM.jpg]
