# Supplementary material for: Glycemic control in the context of frailty: a mortality risk assessment in older diabetic patients
Source: Aging Clin Exp Res. 2026 Apr 9;38(1):129. doi: 10.1007/s40520-026-03385-5 (PMC13194240; doi:10.1007/s40520-026-03385-5)
Supplement: Supplementary file 2 — Supplementary file2 (DOCX 381 kb) [file 40520_2026_3385_MOESM2_ESM.docx]

**Supplementary materials**

**Supplementary Table 1**. Comparison in characteristics between included and excluded sample

| **Characteristics** | **Included (n = 27200)** | **Excluded (n = 1517)** | **Overall (N=28717)** | ***p*-value**^†^ |
| --- | --- | --- | --- | --- |
| **Sex** |  |  |  |  |
| Female | 14386 (52.9%) | 802 (52.9%) | 15188 (52.9%) | 1 |
| Male | 12814 (47.1%) | 715 (47.1%) | 13529 (47.1%) |  |
| **Age, *years*** |  |  |  |  |
| Median [Q1, Q3] | 78.0 [71.0, 84.0] | 83.0 [76.0, 88.0] | 78.0 [71.0, 84.0] | <0.001 |
| 65-74 years | 9995 (36.7%) | 331 (21.8%) | 10326 (36.0%) |  |
| 75-84 years | 11180 (41.1%) | 552 (36.4%) | 11732 (40.9%) |  |
| **≥** 85 years | 6025 (22.2%) | 634 (41.8%) | 6659 (23.2%) | <0.001 |
| **Old-age home residents** |  |  |  |  |
| No | 23547 (86.6%) | 1021 (67.3%) | 24568 (85.6%) | <0.001 |
| Yes | 3653 (13.4%) | 496 (32.7%) | 4149 (14.4%) |  |
| **Season of admission** |  |  |  |  |
| Spring | 7386 (27.2%) | 387 (25.5%) | 7773 (27.1%) |  |
| Summer | 6330 (23.3%) | 315 (20.8%) | 6645 (23.1%) |  |
| Fall | 6155 (22.6%) | 365 (24.1%) | 6520 (22.7%) | 0.103 |
| Winter | 7329 (26.9%) | 450 (29.7%) | 7779 (27.1%) |  |
| **Payment source** |  |  |  |  |
| No public assistance | 17675 (65.0%) | 869 (57.3%) | 18544 (64.6%) | <0.001 |
| Public assistance | 9525 (35.0%) | 648 (42.7%) | 10173 (35.4%) |  |
| **Length of stay, *days*** |  |  |  |  |
| Median [Q1, Q3] | 3.00 [2.00, 6.00] | 4.00 [2.00, 9.00] | 3.00 [2.00, 6.00] | <0.001 |
| **ICD-10 diagnostic codes** |  |  |  |  |
| E10 | 177 (0.7%) | 5 (0.3%) | 182 (0.6%) | 0.308 |
| E11 | 27023 (99.3%) | 1512 (99.7%) | 28535 (99.4%) |  |
| **Hypoglycemia^*^** |  |  |  |  |
| No | 26469 (97.3%) | 1468 (96.8%) | 27937 (97.3%) | 0.449 |
| Yes | 731 (2.7%) | 49 (3.2%) | 780 (2.7%) |  |
| **Cardiovascular diseases^*^** |  |  |  |  |
| No | 24351 (89.5%) | 1276 (84.1%) | 25627 (89.2%) | <0.001 |
| Yes | 2849 (10.5%) | 241 (15.9%) | 3090 (10.8%) |  |
| **Frailty risk^*^** |  |  |  |  |
| No (HFRS = 0) | 16966 (62.4%) | 722 (47.6%) | 17688 (61.6%) | <0.001 |
| Mild (HFRS <5) | 7954 (29.2%) | 555 (36.6%) | 8509 (29.6%) |  |
| Moderate and severe (HFRS ≥5) | 2280 (8.4%) | 240 (15.8%) | 2520 (8.8%) |  |

Characteristics were presented as n (%) for categorical variables and as median [quartile 1, quartile 3] for continuous variables due to skewed distribution. Excluded participants had no time-weight mean HbA1c.

^*^Frailty risk was evaluated utilizing the Hospital Frailty Risk Score, which considers all hospitalization diagnoses occurring within the two years preceding the index hospitalization. Instances of hypoglycemia and cardiovascular diseases were also identified during this timeframe.

^†^The difference among three frailty status groups were assessed using the Wilcoxon test for continuous variables and Chi-square test for categorical variables, respectively.

HFRS: Hospital Frailty Risk Score; ICD-10: International Classification of Diseases, 10th Revision; HbA1c: Hemoglobin A1c.

**Supplementary Table 2**. List of 109 ICD-10 codes to calculate the hospital frailty risk score

| ICD-10 Code | Diagnosis description | Weight |
| --- | --- | --- |
| F00 | Dementia in Alzheimer's disease | 7.1 |
| G81 | Hemiplegia | 4.4 |
| G30 | Alzheimer's disease | 4 |
| I69 | Sequelae of cerebrovascular disease (secondary codes) | 3.7 |
| R29 | Other symptoms/signs involving nervous/musculoskeletal systems (incl. falls) | 3.6 |
| N39 | Other disorders of urinary system (incl. UTI & incontinence) | 3.2 |
| F05 | Delirium, not substance-induced | 3.2 |
| W19 | Unspecified fall | 3.2 |
| S00 | Superficial injury of head | 3.2 |
| R31 | Unspecified hematuria | 3 |
| B96 | Other bacterial agents as cause of diseases (secondary code) | 2.9 |
| R41 | Other symptoms/signs involving cognitive functions | 2.7 |
| R26 | Abnormalities of gait and mobility | 2.6 |
| I67 | Other cerebrovascular diseases | 2.6 |
| R56 | Convulsions, not elsewhere classified | 2.6 |
| R40 | Somnolence, stupor and coma | 2.5 |
| T83 | Complications of genitourinary prosthetic devices | 2.4 |
| S06 | Intracranial injury | 2.4 |
| S42 | Fracture of shoulder and upper arm | 2.3 |
| E87 | Other disorders of fluid/electrolyte/acid-base balance | 2.3 |
| M25 | Other joint disorders, not elsewhere classified | 2.3 |
| E86 | Volume depletion | 2.3 |
| R54 | Senility | 2.2 |
| Z50 | Care involving rehabilitation procedures | 2.1 |
| F03 | Unspecified dementia | 2.1 |
| W18 | Other fall on same level | 2.1 |
| Z75 | Problems related to medical facilities | 2 |
| F01 | Vascular dementia | 2 |
| S80 | Superficial injury of lower leg | 2 |
| L03 | Cellulitis | 2 |
| H54 | Blindness and low vision | 1.9 |
| E53 | Deficiency of other B vitamins | 1.9 |
| Z60 | Problems related to social environment | 1.8 |
| G20 | Parkinson's disease | 1.8 |
| R55 | Syncope and collapse | 1.8 |
| S22 | Fracture of rib(s), sternum and thoracic spine | 1.8 |
| K59 | Other functional intestinal disorders | 1.8 |
| N17 | Acute kidney injury | 1.8 |
| L89 | Pressure ulcer | 1.7 |
| Z22 | Carrier of infectious disease | 1.7 |
| B95 | Streptococcus/staphylococcus as cause of diseases | 1.7 |
| L97 | Ulcer of lower limb, not elsewhere classified | 1.6 |
| R44 | Other symptoms/signs involving general sensations | 1.6 |
| K26 | Duodenal ulcer | 1.6 |
| I95 | Hypotension | 1.6 |
| N19 | Unspecified kidney failure | 1.6 |
| A41 | Other sepsis | 1.6 |
| Z87 | Personal history of other diseases | 1.5 |
| J96 | Respiratory failure, not elsewhere classified | 1.5 |
| X59 | Exposure to unspecified factor | 1.5 |
| M19 | Other osteoarthritis | 1.5 |
| G40 | Epilepsy | 1.5 |
| M81 | Osteoporosis without pathological fracture | 1.4 |
| S72 | Hip fracture | 1.4 |
| S32 | Fracture of lumbar spine and pelvis | 1.4 |
| E16 | Other disorders of pancreatic endocrine function | 1.4 |
| R94 | Abnormal results of function studies | 1.4 |
| N18 | Chronic kidney disease | 1.4 |
| R33 | Urinary retention | 1.3 |
| R69 | Unknown/unspecified causes of morbidity | 1.3 |
| N28 | Other disorders of kidney/ureter | 1.3 |
| R32 | Unspecified urinary incontinence | 1.2 |
| G31 | Other degenerative diseases of nervous system | 1.2 |
| Y95 | Nosocomial condition | 1.2 |
| S09 | Other/unspecified injuries of head | 1.2 |
| R45 | Symptoms/signs involving emotional state | 1.2 |
| G45 | Transient ischemic attacks | 1.2 |
| Z74 | Problems related to caregiver dependency | 1.1 |
| M79 | Other soft tissue disorders | 1.1 |
| W06 | Fall involving bed | 1.1 |
| S01 | Open wound of head | 1.1 |
| A04 | Other bacterial intestinal infections | 1.1 |
| A09 | Diarrhea/gastroenteritis of presumed infectious origin | 1.1 |
| J18 | Pneumonia, organism unspecified | 1.1 |
| J69 | Aspiration pneumonia | 1 |
| R47 | Speech disturbances | 1 |
| E55 | Vitamin D deficiency | 1 |
| Z93 | Artificial opening status | 1 |
| R02 | Gangrene, not elsewhere classified | 1 |
| R63 | Symptoms/signs concerning food/fluid intake | 0.9 |
| H91 | Other hearing loss | 0.9 |
| W10 | Fall on/from stairs and steps | 0.9 |
| W01 | Fall on same level from slipping/tripping | 0.9 |
| E05 | Hyperthyroidism | 0.9 |
| M41 | Scoliosis | 0.9 |
| R13 | Dysphagia | 0.8 |
| Z99 | Dependence on assistive devices | 0.8 |
| U80 | Penicillin-resistant organisms | 0.8 |
| M80 | Osteoporosis with pathological fracture | 0.8 |
| K92 | Other diseases of digestive system | 0.8 |
| I63 | Cerebral infarction | 0.8 |
| N20 | Kidney/ureteral stones | 0.7 |
| F10 | Alcohol use disorders | 0.7 |
| Y84 | Other medical procedures as cause of adverse reaction | 0.7 |
| R00 | Abnormal heart rhythms | 0.7 |
| J22 | Unspecified acute lower respiratory infection | 0.7 |
| Z73 | Problems related to lifestyle management | 0.6 |
| R79 | Other abnormal lab findings | 0.6 |
| Z91 | Personal history of risk factors | 0.5 |
| S51 | Open wound of forearm | 0.5 |
| F32 | Depressive episode | 0.5 |
| M48 | Spinal stenosis (secondary code only) | 0.5 |
| E83 | Disorders of mineral metabolism | 0.4 |
| M15 | Polyosteoarthritis | 0.4 |
| D64 | Other anemias | 0.4 |
| L08 | Other local skin infections | 0.4 |
| R11 | Nausea and vomiting | 0.3 |
| K52 | Other non-infectious gastroenteritis/colitis | 0.3 |
| R50 | Fever of unknown origin | 0.1 |

**Note**:

The 109 ICD-10 codes and their associated weights were derived from the study conducted by Gilbert T. et al. (2018). The calculation of hospital frailty risk score primarily focused on cognitive impairment, functional dependence, falls and fractures, anxiety and depression, incontinence, pressure ulcers, and mobility issues.

Gilbert T, Neuburger J, Kraindler J, et al. Development and validation of a Hospital Frailty Risk Score focusing on older people in acute care settings using electronic hospital records: an observational study. Lancet. 2018;391(10132):1775-1782. doi:10.1016/S0140-6736(18)30668-8.

**Supplementary Table 3**. Linear association between time-weighted mean HbA1c and mortality by frailty risk among older adults with diabetes

| **Models*** | **Overall** | |  | **No**  **(HFRS = 0)** | |  | **Mild**  **(HFRS <5)** | |  | **Moderate and severe**  **(HFRS ≥5)** | | ***p*-value for interaction**^†^ |
| --- | --- | --- | --- | --- | --- | --- | --- | --- | --- | --- | --- | --- |
|  | Hazard ratio (95% confidence interval) | *p*-value |  | Hazard ratio (95% confidence interval) | *p*-value |  | Hazard ratio (95% confidence interval) | *p*-value |  | Hazard ratio (95% confidence interval) | *p*-value |  |
| Model 1 | 1.043 (1.030–1.056) | <0.001 |  | 1.058 (1.040–1.076) | <0.001 |  | 1.015 (0.993–1.037) | 0.177 |  | 1.058 (1.020–1.099) | 0.003 | 0.009 |
| Model 2 | 1.048 (1.035–1.061) | <0.001 |  | 1.062 (1.044–1.080) | <0.001 |  | 1.021 (1.000–1.044) | 0.054 |  | 1.059 (1.020–1.100) | 0.003 | 0.008 |
| Model 3 | 1.046 (1.033–1.060) | <0.001 |  | 1.058 (1.040–1.077) | <0.001 |  | 1.022 (1.000–1.045) | 0.046 |  | 1.060 (1.020–1.101) | 0.003 | 0.010 |

^*^The linear association between time-weighted mean HbA1c and mortality was evaluated using Cox regression analysis. Model 1 was unadjusted except for frailty status in the overall population. Model 2 included adjustments for age, sex, residence in an old-age home, and payment source at the time of index hospitalization. Model 3 further adjusted for the season of admission, length of stay during the index hospitalization, as well as instances of hypoglycemia and cardiovascular events occurring within two years prior to the index hospitalization.

^†^The *p*-value for interaction was assessed using a likelihood ratio test, which compared Cox regression models with and without the interaction term for time-weighted mean HbA1c and frailty risk group.

Frailty risk was evaluated utilizing the Hospital Frailty Risk Score (HFRS), which considers all hospitalization diagnoses occurring within the two years preceding the index hospitalization. Instances of hypoglycemia and cardiovascular diseases were also identified during this timeframe.

**Supplementary Table 4**. Linear association between overall mean HbA1c and mortality by frailty risk among older adults with diabetes

| **Models*** | **Overall** | |  | **No**  **(HFRS = 0)** | |  | **Mild**  **(HFRS <5)** | |  | **Moderate and severe**  **(HFRS ≥5)** | | ***p*-value for interaction**^†^ |
| --- | --- | --- | --- | --- | --- | --- | --- | --- | --- | --- | --- | --- |
|  | Hazard ratio (95% confidence interval) | *p*-value |  | Hazard ratio (95% confidence interval) | *p*-value |  | Hazard ratio (95% confidence interval) | *p*-value |  | Hazard ratio (95% confidence interval) | *p*-value |  |
| Model 1 | 1.041 (1.028**–**1.055) | <0.001 |  | 1.059 (1.041**–**1.078) | <0.001 |  | 1.011 (0.990**–**1.033) | 0.306 |  | 1.049 (1.011**–**1.090) | 0.012 | 0.005 |
| Model 2 | 1.045 (1.031**–**1.058) | <0.001 |  | 1.061 (1.043**–**1.080) | <0.001 |  | 1.017 (0.995**–**1.039) | 0.137 |  | 1.048 (1.009**–**1.088) | 0.016 | 0.004 |
| Model 3 | 1.043 (1.030**–**1.056) | <0.001 |  | 1.058 (1.039**–**1.077) | <0.001 |  | 1.017 (0.995**–**1.038) | 0.128 |  | 1.048 (1.009**–**1.089) | 0.016 | 0.005 |

^*^The linear association between overall mean HbA1c and mortality was evaluated using Cox regression analysis. Model 1 was unadjusted except for frailty status in the overall population. Model 2 included adjustments for age, sex, residence in an old-age home, and payment source at the time of index hospitalization. Model 3 further adjusted for the season of admission, length of stay during the index hospitalization, as well as instances of hypoglycemia and cardiovascular events occurring within two years prior to the index hospitalization.

^†^The *p*-value for interaction was assessed using a likelihood ratio test, which compared Cox regression models with and without the interaction term for overall mean HbA1c and frailty risk group.

Frailty risk was evaluated utilizing the Hospital Frailty Risk Score (HFRS), which considers all hospitalization diagnoses occurring within the two years preceding the index hospitalization. Instances of hypoglycemia and cardiovascular diseases were also identified during this timeframe.


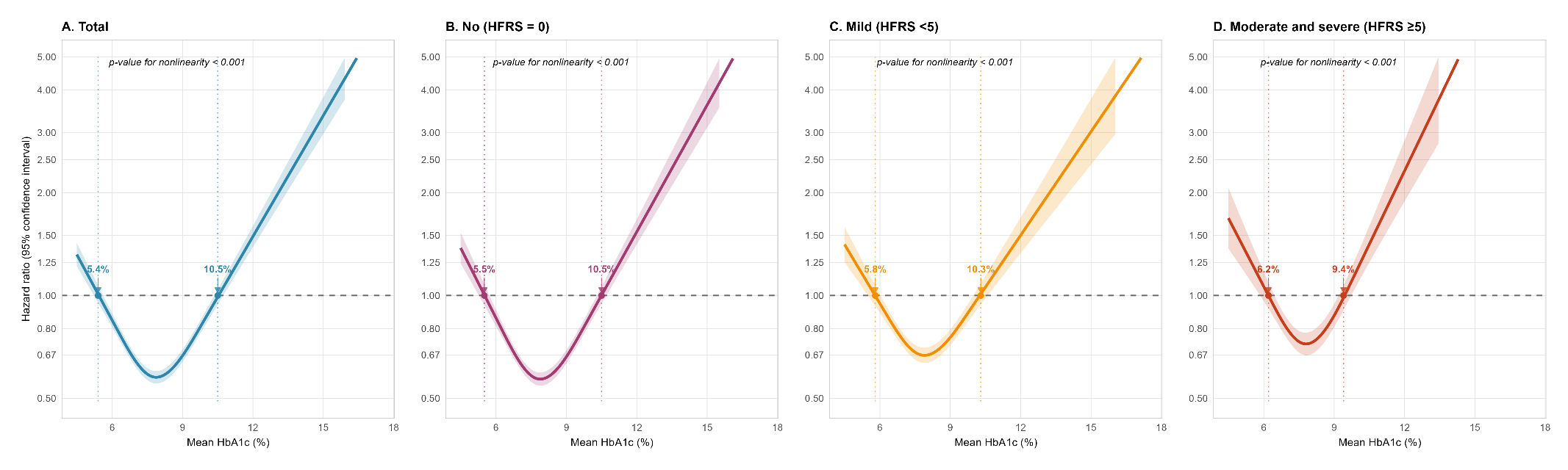


**Supplementary Figure 1**. The restricted cubic spline curve for the nonlinear association between overall mean HbA1c and mortality by frailty risk among older adults with diabetes

**Note:**

**A**. Total patients; **B**. Patients without frailty risk (HFRS = 0); **C**. Patients with mild frailty risk (HFRS <5); **D**. Patients with moderate and severe frailty risk (HFRS ≥5)

Restricted cubic spline curve was fitted with three knots at the 10th, 50th, and 90th percentiles using Cox regression. This approach was applied both to the overall population and within specific frailty risk subgroups. The *p*-value for nonlinearity was derived from a Wald Chi-square test.

The intersection points on the spline curve represent values of overall mean HbA1c where the hazard ratio equals 1. These points indicate the thresholds at which the predicted mortality risk aligns statistically with the reference risk level.

The Cox regression model was adjusted for a variety of covariates, including age, sex, residence in old-age home, payment source, season of admission, length of stay during the index hospitalization, and instances of hypoglycemia and cardiovascular events occurring within two years prior to the index hospitalization.

Frailty risk was evaluated utilizing the Hospital Frailty Risk Score (HFRS), which considers all hospitalization diagnoses occurring within the two years preceding the index hospitalization. Instances of hypoglycemia and cardiovascular diseases were also identified during this timeframe.
